# Modeling immersion pathways in XR-based cultural heritage IP narrative experiences: an integrated approach based on TAM, experience economy, and grounded theory

**DOI:** 10.3389/fpsyg.2026.1818614

**Published:** 2026-06-10

**Authors:** Dan Liu, Jungho Jung

**Affiliations:** 1 Interdisciplinary Program of Arts & Design Technology, Chonnam National University, Gwangju, Republic of Korea; 2 School of Information Engineering, Liaodong University, Dandong, China; 3 Department of Design, Chonnam National University, Gwangju, Republic of Korea

**Keywords:** behavioral intention, cultural Value Identification, cultural-heritage storytelling, Emotional Arousal, experiential value (4E), mobile XR, narrative design, Psychological Immersion

## Abstract

**Introduction:**

Mobile extended reality (XR) heritage storytelling is increasingly experienced on smartphones in short, interruptible sessions, where behavioral intention may be shaped by immediate in-use responses rather than extended post-experience reflection. Using the Great Wall E-tour (云游长城) WeChat Mini Program as the research context, this study examines how technical appraisal, narrative design, and experiential value are associated with immediate post-experience behavioral intention through affective, cultural, and immersion-related mechanisms.

**Methods:**

We adopted an exploratory sequential mixed-methods design. A grounded-theory-informed qualitative phase provided experience-near support for the mechanism account and helped contextualize the constructs. This was followed by a post-experience survey analyzed with confirmatory factor analysis, structural equation modeling, and bias-corrected bootstrapped mediation tests.

**Results:**

XR Technology Perception, Narrative Design, and Experiential Value were positively associated with Emotional Arousal and Cultural Value Identification. In the theory-specified model, Emotional Arousal and Cultural Value Identification were also associated with Psychological Immersion. At the outcome stage, Emotional Arousal showed the most consistent direct association with Behavioral Intention, while Cultural Value Identification was significant in the theory-specified model but less stable in the expanded direct-effects specification. Psychological Immersion showed a weaker and more model-sensitive role. Supplementary checks, including common-method-bias diagnostics, XTP measurement-model comparison, prior-XR-experience analyses, and alternative structural models, suggest that Emotional Arousal, Cultural Value Identification, and Psychological Immersion should be understood as closely related mechanisms rather than as a fixed causal chain.

**Discussion:**

From a media-psychology and human-factors design perspective, mobile immersive heritage systems should prioritize emotionally engaging cues, meaningful story–task structures, low-friction interaction, and clear action bridges, while treating immersion as support for involvement rather than the sole route to intention formation.

## 1 Introduction

Digital technologies increasingly shape how people attend to, interpret, and act on information in everyday life. In mobile, interactive settings, user responses are often shaped less by “what is shown” than by how the system supports attention, control, and moment-to-moment engagement. Users make fast judgments about whether an experience is worth continuing, sharing, or returning to, based on perceived usefulness and perceived ease of use (Davis, 1989), as well as interaction cues such as responsiveness, feedback clarity, and usability (Capece et al., 2024; Lee et al., 2020; Zheng et al., 2024). Evidence from post-adoption and engagement research likewise shows that perceived interface and interaction quality is closely tied to continuance and sustained engagement in technology-mediated services (O'Brien and Toms, 2010; Wixom and Todd, 2005). This mechanism is particularly salient in short, interruptible mobile sessions, where decisions to proceed or drop out are made quickly and are tightly coupled with immediate psychological responses.

Extended reality (XR) and interactive digital storytelling are increasingly used to support cultural-heritage experiences that encourage intention-related responses, such as continued engagement, sharing, recommendation, and follow-up learning—via digitally mediated sessions (Bekele et al., 2018; Innocente et al., 2023; Okanovic et al., 2022; Shim et al., 2024). Importantly, mobile platforms often package XR heritage content as short, interruptible sessions that rely on lightweight participation: guided choices, micro-missions, task completion, and feedback loops. Recent reviews and design surveys suggest that these “task-based narrative” patterns are common in mobile cultural experiences because they help secure attention quickly and provide a clear progression structure for meaning-making (Marques et al., 2023; Nikolarakis and Koutsabasis, 2024; Silva et al., 2023; Wang et al., 2024). Under these conditions, the core design question becomes behavioral: what helps translate a brief mobile XR encounter into intentions to continue, recommend, or visit offline?

Immersion is often treated as the main route through which experience relates to intention. Yet in mobile, light-interaction settings, users may reach momentary emotional peaks more easily than they sustain a stable immersive state. This makes it essential to examine proximal psychological mechanisms that arise early and may shape later intention. Two mechanisms are especially relevant for heritage storytelling. Emotional arousal (EA) captures immediate affective activation triggered by narrative cues, interactivity, and feedback. Cultural value identification (CVI) captures a value-laden appraisal through which cultural meanings are acknowledged, endorsed, or seen as worth sharing. Narrative design can plausibly activate both mechanisms through character resonance, plot progression, and interpretive scaffolding (Jenkins, 2006; Murray, 2017; Ryan, 2015). Likewise, work in digital heritage and museum settings indicates that usability, responsiveness, and perceived interaction quality are linked to experience quality and continuance tendencies (Capece et al., 2024; Davis, 1989; Lee et al., 2020; Zheng et al., 2024). The experience economy framework organizes experiential value into Entertainment, Education, Esthetics, and Escapism (Pine and Gilmore, 2011). These dimensions have been used to explain post-experience intentions in cultural and visitor settings (Bassano et al., 2019; Mehmetoglu and Engen, 2011; Moreira et al., 2024; Oh et al., 2007).

Despite rapid growth, existing work still often relies on single-factor explanations or treats key mechanisms in isolation. Few studies have integrated technical appraisal, narrative organization, and experiential value within a single structural framework. As a result, the relative roles of these design and experience domains in emotional arousal (EA), cultural value identification (CVI), psychological immersion (IM), and behavioral intention (BI) remain underspecified. Prior research on online VR exhibition experiences suggests that immersion-related experience can be associated with users’ acceptance and continued-use intentions, but the relative role of immersion versus more proximal mechanisms remains underspecified (Li and Lv, 2024). This leaves two practical uncertainties for mobile XR heritage design: (i) under short-session constraints, do EA and CVI function as more stable near-term routes to BI? and (ii) does IM operate as the main route to BI, or as an experience-deepening add-on to EA/CVI?

To address these issues, this study proposes an Integrated Immersion Pathway Model for mobile XR cultural-heritage IP narrative experiences. We operationalize XR Technology Perception (XTP) as a context-specific holistic technical appraisal of the mobile XR experience. It is indicated by perceived XR system/interaction cues and two TAM belief items: perceived usefulness (PU) and perceived ease of use (PEOU). At the same time, we explicitly acknowledge that these components remain conceptually distinguishable in classical information-systems research. XTP is modeled alongside Narrative Design (ND) and Experiential Value (4E), the latter treated as an overall experiential-value appraisal informed by Entertainment, Education, Esthetics, and Escapism. EA and CVI are specified as proximal psychological mechanisms, and IM is positioned as a theory-specified experience-deepening state associated with EA/CVI and BI. Because the data are cross-sectional and collected immediately after the experience, this ordering is not interpreted as a definitive temporal sequence. Empirically, we test both single-step pathways (antecedent → EA/CVI → BI) and theory-specified IM-related indirect routes (antecedent → EA/CVI → IM → BI), while also reporting supplementary alternative models that examine reverse and parallel psychological ordering.

This study makes three contributions. First, it extends media-psychological research on interactive and narrative experiences by modeling how short-session mobile XR heritage storytelling translates design antecedents into immediate post-experience behavioral intentions. Second, it advances mechanism-based explanation by examining EA, CVI, and IM as a closely related psychological mechanism cluster rather than assuming that immersion is the sole or automatically dominant converter of experience into intention. Third, the study integrates TAM-based technical appraisal, the experience economy framework, and qualitative coding informed by grounded-theory procedures within a single structural model. This integration clarifies the relative roles of technical appraisal, narrative-task organization, and experiential cues in shaping affective and value-based processing. Together, this framework provides a theoretically grounded but appropriately bounded basis for designing story–task flows, feedback, and experience cues that support behavior-relevant outcomes in everyday, short-session use.

## 2 Literature review and hypotheses development

### 2.1 XR Technology Perception in mobile heritage storytelling (XTP)

Mobile XR heritage storytelling is typically encountered on smartphones in brief, interruptible sessions. In this setting, users often form rapid technical judgments early in the interaction, based on cues such as responsiveness, controllability, feedback clarity, interface consistency, and stability. These judgments shape whether the experience feels effortless enough to continue and structured enough to be worth attention. System/interaction quality, perceived usefulness, and perceived ease of use remain theoretically distinct. In short, interruptible mobile sessions, users may nevertheless integrate these cues into an immediate technical appraisal.

The classic Technology Acceptance Model (TAM) explains adoption through perceived usefulness (PU) and perceived ease of use (PEOU) (Davis, 1989). Information-systems research also distinguishes system quality, service/interface quality, and user beliefs as conceptually separate components with different antecedent–consequent networks (DeLone and McLean, 2003; Wixom and Todd, 2005). In the present short-session mobile XR context, we therefore do not treat PU/PEOU and XR system/interaction cues as interchangeable constructs in general. Instead, we use them to represent a context-specific user appraisal of whether the technical experience is smooth, understandable, useful, and easy enough to continue.

In this study, XR Technology Perception (XTP) is defined as a context-specific holistic technical appraisal of the mobile XR session. This definition preserves the conceptual distinction among XR system/interaction cues, PU, and PEOU while recognizing that, in a brief story–task interaction, users may experience these cues as a coherent technical-entry condition. XTP is therefore expected to influence proximal mechanisms—emotional arousal (EA) and cultural value identification (CVI)—by shaping whether users can move smoothly through the story–task flow and remain receptive to narrative and experiential cues.

*H1a*: XTP positively influences EA.

*H1b*: XTP positively influences CVI.

### 2.2 Narrative design in mobile XR heritage storytelling (ND)

In mobile XR heritage storytelling, users do not experience “story content” alone; they experience a story–task sequence that organizes attention, action, and interpretation within a limited time window. Narrative design (ND) therefore functions as a behavioral structure: it shapes what users notice first, what they do next, and how meaning is assembled across role framing, micro-missions, and feedback. When progression is coherent and guidance is clear, users are more likely to stay with the flow, reach emotional peaks at key moments, and form interpretable cultural takeaways rather than fragmented impressions.

Media-psychological accounts emphasize that coherent narrative processing and guided transportation support emotional engagement and meaning-focused processing (Busselle and Bilandzic, 2009; Green and Brock, 2000). Interactive storytelling perspectives likewise highlight role-based participation, paced revelation, and structured progression as drivers of engagement (Dow et al., 2007; Jenkins, 2006; Murray, 2017; Ryan, 2015). Under short-session conditions, these design properties matter even more because users have less time to “settle into” the story. ND therefore needs to reduce ambiguity quickly, sustain momentum, and make next actions legible.

In the proposed model, ND is treated as a parallel upstream domain to XTP and 4E. A coherent story–task structure is expected to heighten EA through role resonance and paced progression, and to strengthen CVI by scaffolding interpretation—helping users translate cultural symbols and messages into personally endorsable meaning rather than merely “information encountered.”

*H2a*: ND positively influences EA.

*H2b*: ND positively influences CVI.

### 2.3 Experiential value in mobile XR storytelling (4E)

Alongside XTP and ND, experiential value captures how the session feels during use and what it affords psychologically in the moment. The experience-economy framework conceptualizes experiential value through four theoretically distinct but complementary dimensions: Entertainment, Education, Esthetics, and Escapism (4E) (Pine and Gilmore, 2011). It emphasizes that experience emerges from multiple cues rather than a single post-hoc satisfaction judgment. In tourism and cultural experience research, these dimensions have been linked to post-experience outcomes such as evaluation, memory-related responses, and behavioral intention (Mehmetoglu and Engen, 2011; Oh et al., 2007).

In short-session mobile XR storytelling, 4E is understood as an integrated set of immediately perceived experiential cues embedded in the interaction flow. Entertainment and escapism cues are conveyed through task progression, micro-challenges, and feedback loops; education cues through narration and interpretive prompts; and esthetics cues through scene atmosphere, audiovisual coherence, and interface presentation. Under brief and interruptible conditions, users may form a rapid overall appraisal of the experience rather than stable dimension-by-dimension judgments. Therefore, 4E is operationalized in this study as an overall experiential-value appraisal informed by four theoretically distinct dimensions, rather than as a claim that the four dimensions are interchangeable in all contexts. This modeling choice is evaluated empirically in the Results section.

Stronger perceived experiential value is expected to facilitate intention formation primarily through proximal mechanisms that arise during use—namely EA and CVI. Emotionally engaging cues and coherent aesthetic presentation can increase affective activation, while interpretive and meaning-support cues can strengthen identification with the cultural values conveyed by the narrative. Thus, 4E is positioned as an upstream experiential domain predicting EA and CVI.

*H3a*: 4E positively influences EA.

*H3b*: 4E positively influences CVI.

### 2.4 Proximal mechanisms: emotional arousal (EA) and cultural value identification (CVI)

In mobile, light-interaction settings, mediated-message processing is constrained by limited attentional and cognitive resources (Lang, 2000). Users may therefore reach emotional peaks and recognize cultural value more quickly than they sustain a durable immersive state. We treat EA and CVI as proximal psychological mechanisms that can emerge early in short-session mobile XR experiences and relate to immediate post-experience behavioral intention.

Emotional arousal (EA) refers to heightened affective activation during the session, such as feeling moved or impressed. Emotion is not only a post-hoc residue of experience. It can also prioritize attention and increase approach tendencies toward salient stimuli (Picard, 2000; Vuilleumier, 2005). In brief mobile XR sessions, continuation and sharing decisions can occur quickly. EA may therefore help translate design cues into immediate intention, even when a stable immersive state has not fully formed.

Cultural value identification (CVI) captures whether users recognize, endorse, and internalize the cultural meaning conveyed by the heritage content. It reflects whether the experience feels aligned with one’s values and worth communicating. The construct is informed by social identification research, which emphasizes internalized meaning and self-relevance as routes through which content becomes motivation and intention (Ashforth and Mael, 1989). It is also grounded in heritage interpretation theory. Classic interpretation scholarship argues that heritage communication should not simply transmit factual information; it should help audiences connect heritage meanings with their own experiences, values, and sense of relevance (Ham, 2016; Tilden, 2009). In the present mobile XR context, CVI refers to users’ immediate value-based recognition of cultural meaning in the story–task experience. It provides a rationale for recommending, sharing, or following up offline because the heritage meaning is perceived as personally relevant and worth carrying forward.

### 2.5 Psychological immersion as an experience-deepening mechanism (IM)

In this study, Psychological Immersion (IM) refers to a subjective state of attentional absorption and felt involvement in the mobile XR story–task flow. It is related to presence, flow, and narrative transportation, but it is not treated as identical to any of these concepts. Nor does IM refer to objective technological immersiveness or system-level XR capability. Rather, it captures the extent to which users feel mentally absorbed, involved in the unfolding task, and less aware of the surrounding environment during a brief smartphone-based heritage experience. This distinction is important because the present study examines mobile XR in a short, interruptible session, not a fully immersive HMD-based virtual environment.

Prior research on immersive and interactive media has distinguished enabling system conditions from users’ experienced psychological involvement. Concentrated attention, cognitive resource allocation, and subjective absorption are central to the experience of immersion, even when the technological environment itself varies in its degree of immersiveness (Slater and Wilbur, 1997; Witmer and Singer, 1998). Similarly, game and interactive media research has treated immersion as a user-level psychological state that can be assessed through perceived involvement and absorption (Jennett et al., 2008). In this sense, IM in the present study is positioned as an experience-deepening mechanism within the story–task process.

The proposed model specifies EA and CVI as proximal psychological responses associated with IM. Emotional arousal may heighten attentional priority and provide motivational energy for users to stay with the unfolding experience, consistent with emotion research linking affective appraisal to action readiness and motivational tendency (Frijda et al., 1989; Vuilleumier, 2005). Cultural value identification may also deepen involvement by giving the narrative task personal relevance and interpretive meaning. This specification, however, should not be read as a fixed temporal sequence. In short-session mobile XR experiences, emotional activation, cultural meaning, and immersion may emerge closely together, and immersion may also reinforce emotional engagement or cultural meaning processing. Therefore, the EA/CVI → IM paths are treated as theoretically specified associations within the proposed model rather than as definitive causal ordering.

*H4a*: EA positively influences IM.

*H4b*: CVI positively influences IM.

IM may further relate to behavioral intention by helping users sustain involvement, complete the story–task sequence, and consolidate their experience impressions. Prior work on immersion and interactive experience suggests that subjective immersion involves attentional absorption and task involvement, which can make the experience more salient and easier to carry forward after use (Jennett et al., 2008). Related narrative-transportation research also indicates that absorption into a story can shape evaluations, beliefs, and story-consistent responses, although IM in this study is not treated as identical to transportation (Green and Brock, 2000). In immersive VR research, immersion has also been linked to affective and cognitive value pathways that support perceived outcomes and motivation (Makransky and Lilleholt, 2018; Qiu et al., 2024). Still, short mobile sessions may not allow immersion to stabilize into a strong and durable state. Its direct relationship with immediate post-experience BI may therefore be weaker and more sensitive to model specification than the more immediate routes through EA and CVI.

*H5*: IM positively influences BI.

### 2.6 Integrated model and hypotheses summary

The proposed model is related to the stimulus–organism–response (S-O-R) tradition, which explains how environmental or media stimuli are linked to behavioral responses through internal psychological states (Mehrabian and Russell, 1974). A broad S-O-R frame, however, does not specify which design antecedents matter most in short-session mobile XR heritage storytelling. It also does not distinguish how affective, cultural, and immersion-related mechanisms operate in this setting. The present model retains the mediated-response logic of S-O-R while specifying three experience antecedents—XTP, ND, and 4E—and three mechanism states—EA, CVI, and IM.

The model is also adjacent to task–technology fit (TTF) perspectives, which explain technology use through the fit between task requirements and technological functions (Goodhue and Thompson, 1995). TTF is useful for understanding whether a digital heritage system supports task completion. Recent digital museum research has also used TTF-related perspectives to explain continuance intention (Zheng et al., 2024). However, the Great Wall E-tour is not only a functional task system. It combines technical interaction, restoration tasks, narrative guidance, and cultural interpretation. A mechanism model is therefore needed to explain how these elements relate to emotion, cultural meaning, immersion, and intention.

Thus, the current integrated model does not replace S-O-R or TTF. It adapts their useful insights to a more specific question: how short, interruptible mobile XR heritage experiences link technical appraisal, story–task design, and experiential value to immediate intention through affective, cultural, and involvement-related mechanisms.

Bringing these perspectives together, the proposed model treats XTP, ND, and 4E as three parallel antecedent domains of short-session mobile XR heritage experience. XTP represents users’ holistic technical appraisal, ND captures the perceived organization of the story–task structure, and 4E reflects the overall experiential-value appraisal formed during the session. These upstream domains are expected to relate to immediate post-experience behavioral intention (BI) through a set of closely connected psychological mechanisms: emotional arousal (EA), cultural value identification (CVI), and psychological immersion (IM).

**Figure 1:**
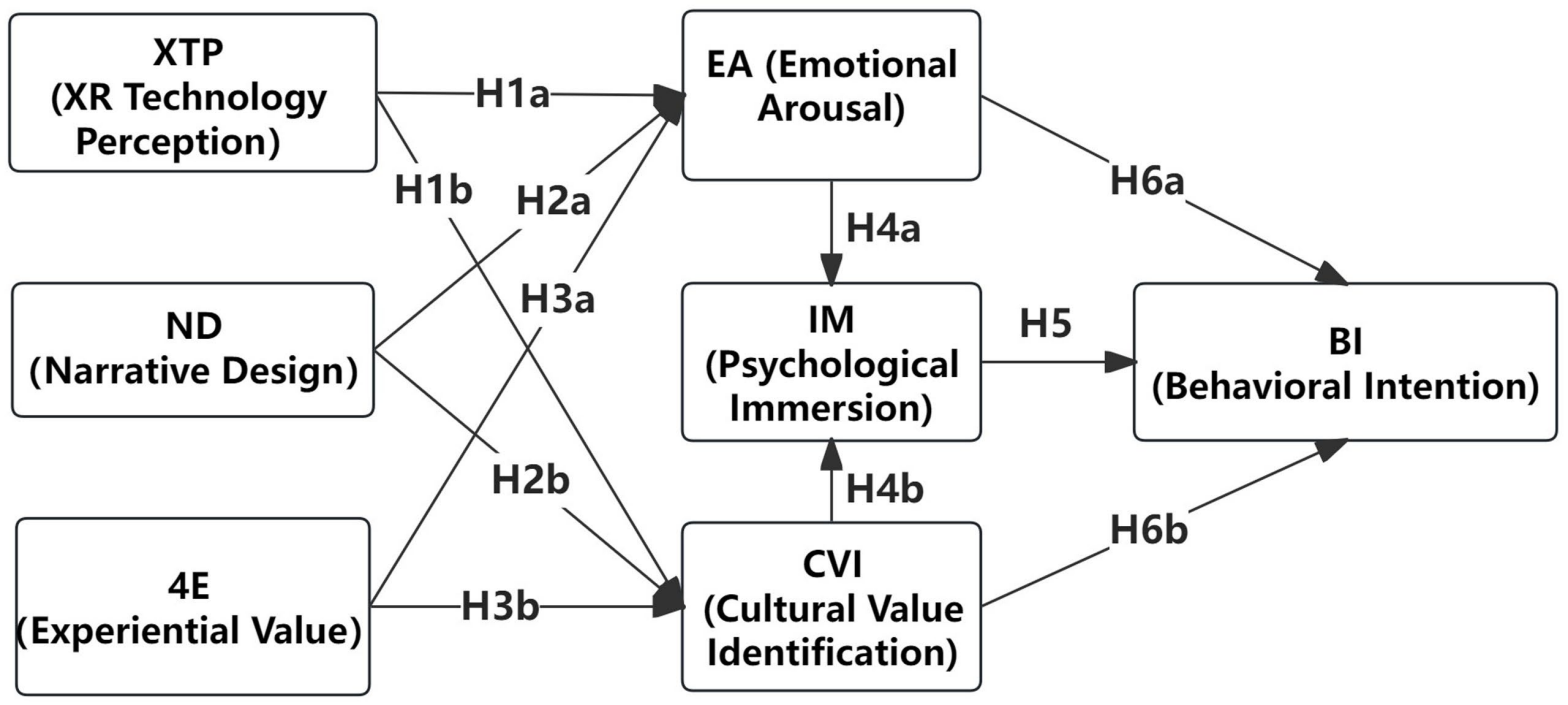
Research model and hypothesized pathways.

In this model, EA and CVI are specified as proximal psychological responses because affective activation and value-based meaning can emerge quickly during a brief mobile session. IM is positioned as a theory-specified experience-deepening state that may further support involvement in the story–task flow. Figure 1 summarizes the research model and hypothesized pathways. The model therefore examines whether immediate BI is more closely associated with affective activation and cultural value identification, while also testing whether IM provides an additional involvement-related pathway.

At the same time, the proposed structure should not be read as a fixed temporal sequence. Because the study relies on a cross-sectional post-experience survey, the model tests theoretically specified associations rather than definitive causal ordering. Direct paths from EA and CVI to BI are retained because short-session intentions may form before a stable immersive state is fully established. EA is expected to relate to BI through heightened action readiness, while CVI is expected to relate to BI through value endorsement and meaning carryover, consistent with affect and identification mechanisms (Ashforth and Mael, 1989; Hosany and Gilbert, 2010).

*H6a*: EA positively influences BI.

*H6b*: CVI positively influences BI.

## 3 Materials and methods

### 3.1 Study context

**Figure 2:**
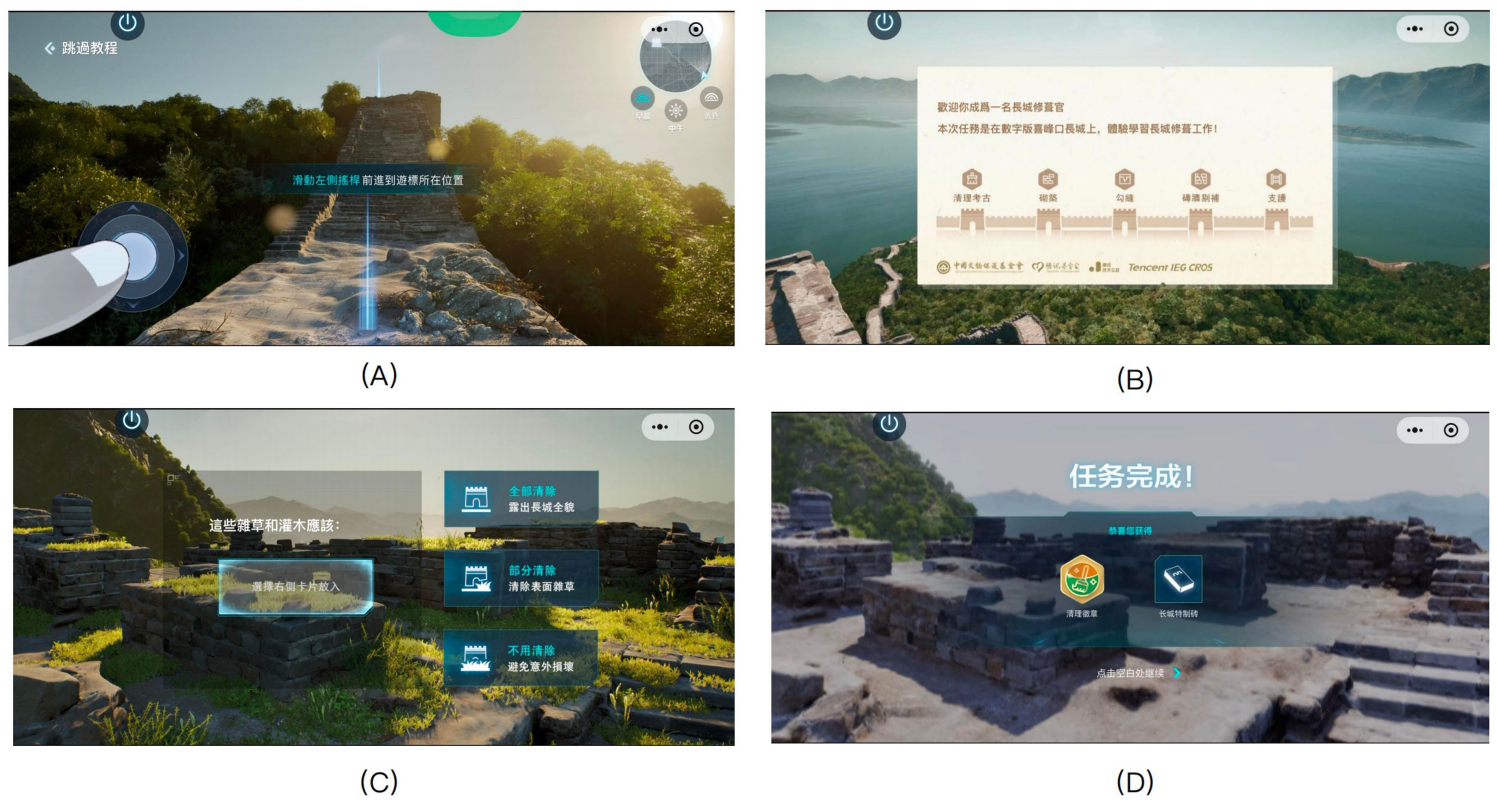
Representative interfaces of the Great Wall E-tour (云游长城) WeChat Mini Program.

The study used the Great Wall E-tour (云游长城) WeChat Mini Program, a smartphone-based mobile XR cultural-heritage storytelling experience organized around interactive restoration tasks. The program’s core interface flow is summarized in Figures 2A–D: onboarding and 3D exploration (A), audio narration (B), interactive restoration tasks (C), and task feedback and guidance (D). Participants completed a standardized 10–20 min story–task session following this sequence (Academy of Chinese Studies, 2025). As a UNESCO World Heritage site inscribed in 1987, the Great Wall provides a relevant heritage context for examining intention formation in short, interruptible mobile XR use (UNESCO World Heritage Centre, 1987). The empirical session was conducted in China with Chinese participants; therefore, CVI in this study should be understood within a domestic cultural-heritage communication context rather than as a cross-cultural reception process.

### 3.2 Research design and procedure

**Figure 3:**
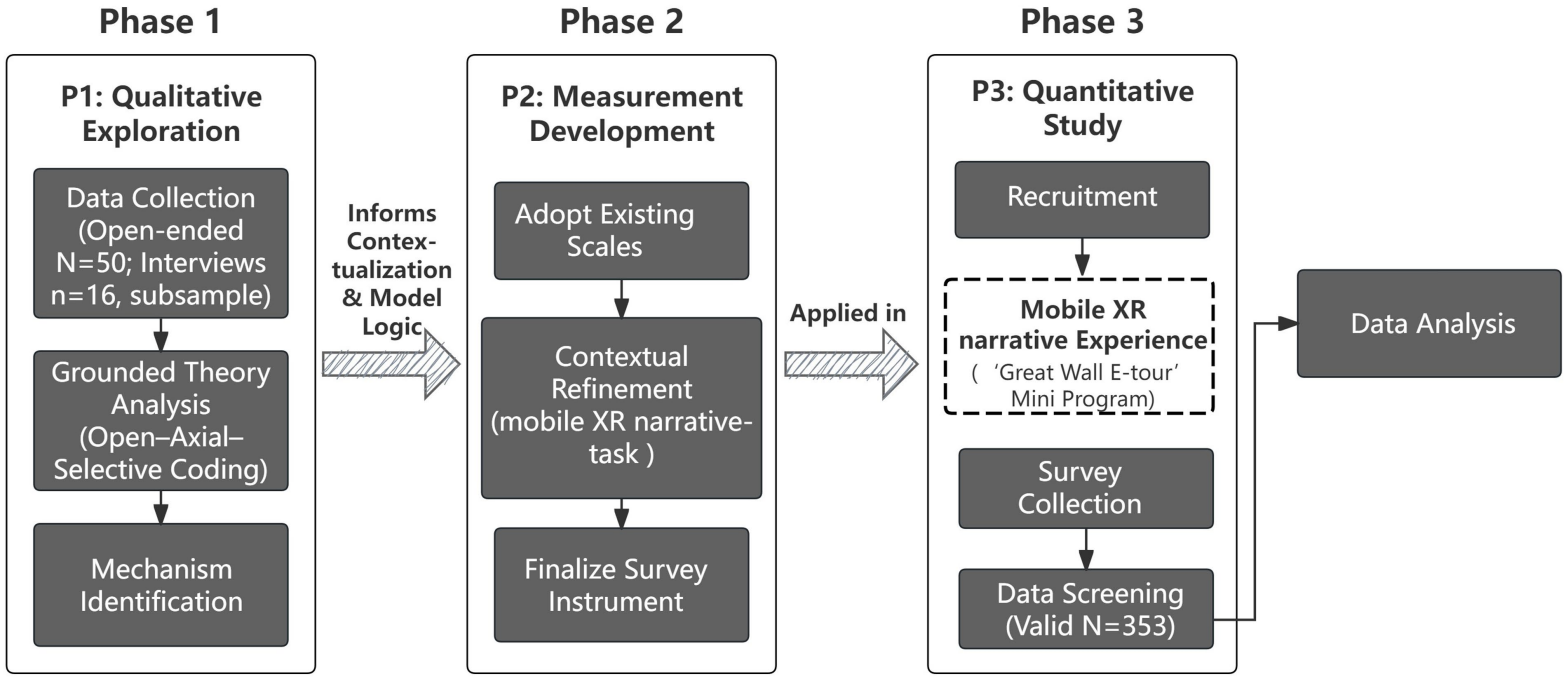
Research design overview.

As shown in Figure 3, we used an exploratory sequential mixed-methods design implemented in three phases. The same structured post-experience questionnaire was used across phases, and Phase 1 survey responses were retained in the final quantitative dataset.

Phase 1 (qualitative exploration) consisted of a post-experience survey completed by 50 participants, with one open-ended prompt appended to the questionnaire. Within this group, 16 participants completed semi-structured interviews. The qualitative materials were analyzed using grounded-theory coding to provide experience-near support for the EA/CVI mechanism account and to inform minor contextual wording refinements. The construct definitions and scale structure were not changed.

Phase 2 (measurement refinement) finalized the survey instrument by incorporating minor contextual wording refinements for the mobile XR narrative-task context. No substantive items were added or removed after Phase 1, and the construct definitions and scale structure remained unchanged.

Phase 3 (quantitative study) implemented an on-site, Institutional Review Board (IRB)-approved smartphone session following a pre-specified 10–20 min protocol, followed immediately by the structured post-experience survey. The Phase 3 dataset was used to evaluate the measurement and structural models and to test the hypothesized direct and indirect effects (see Section 3.6).

### 3.3 Participants

For the quantitative phase, participants were recruited on-site in China through convenience sampling between 1 and 11 October 2025. Participants were eligible if they were able to complete the smartphone-based Great Wall E-tour session and the post-experience questionnaire independently. All participants were Chinese and completed the experience in a Chinese cultural and language context. A total of 364 questionnaires were returned; after excluding incomplete or invalid responses, 353 valid responses were retained (valid response rate = 97.0%). Sample characteristics are summarized in Table 1. The final analytic sample (*N* = 353) included the Phase 1 survey respondents.

**Table 1:** Sample characteristics (*N* = 353).

Variable	Category	Frequency	Percentage (%)
Gender	Male	157	44.5
	Female	196	55.5
Age	18–20	135	38.2
	21–23	117	33.1
	24–25	101	28.6
XR device experience	Frequent	173	49.0
	Occasional	120	34.0
	None	60	17.0

Percentages may not sum to 100 due to rounding.

The sample was relatively homogeneous in educational background and mobile-use familiarity. This profile helped reduce noise attributable to basic operational barriers, but it also narrows the generalizability of the findings and may have contributed to the clean factor structure and high model fit. Accordingly, the findings are best understood as evidence from a Chinese, university-based, mobile-proficient participant sample, and future studies should examine whether the same mechanism pattern holds among broader heritage-tourism populations and international audiences.

### 3.4 Qualitative exploration and coding procedure

#### 3.4.1 Materials and aim

Phase 1 qualitative materials included one open-ended prompt appended to the post-experience survey (*N* = 50) and semi-structured interviews with a subsample (*n* = 16). These materials provided the basis for the qualitative coding reported below.

#### 3.4.2 Coding and model alignment

Qualitative coding was informed by grounded-theory procedures, including open, axial, and selective coding (Charmaz, 2006; Glaser and Strauss, 2017). This analysis developed an experience-near account of how participants described the mobile XR story–task session. The qualitative analysis did not aim to generate a separate theory independent of the quantitative model. Rather, it was used to provide contextual support for the proposed mechanism account and to refine the alignment between measurement items and the mobile XR heritage setting.

During open coding, meaningful expressions related to technical fluency, story guidance, experiential cues, emotional activation, cultural meaning, immersion, and follow-up intention were identified from the open-ended survey responses and interview transcripts. During axial coding, related codes were grouped into higher-level categories, including technical fluency, narrative scaffolding, experiential value, affective response, cultural identification, psychological involvement, and behavioral extension. During selective coding, these categories were further aligned with the SEM constructs: XTP, ND, 4E, EA, CVI, IM, and BI. This procedure helped ensure that the constructs were grounded not only in prior theory and established scales, but also in participants’ actual descriptions of the short-session mobile XR heritage experience.

Table 2 summarizes the coding-to-construct alignment by showing how representative open codes and translated participant excerpts were grouped into axial categories and linked to the SEM constructs. Full coding dictionaries, source identifiers, additional excerpts, and item-level mapping are provided in Supplementary file S3.

**Table 2:** Qualitative coding summary and SEM construct alignment.

Selective theme	Axial category	Representative open codes	Representative participant excerpt (translated)	SEM construct
Technical fluency	Realism, smoothness, feedback clarity	realistic scene; smooth operation; timely response; clear feedback	“The system ran smoothly, and the feedback made it clear what I should do next.” (I03)	XR Technology Perception (XTP)
Narrative scaffolding	Story guidance, task progression, role framing	character guidance; mission logic; story involvement; task progression	“The narrative guidance made the mission clear and helped me follow the task step by step.” (I09)	Narrative Design (ND)
Experiential appraisal	Entertainment, education, esthetics, escapism	fun; learning; beautiful scene; time-travel feeling; role substitution	“I learned a lot about the Great Wall while completing the tasks, and the scenes made the experience more interesting.” (I02)	Experiential Value (4E)
Affective activation	Emotional response and resonance	moved; shocked; impressed; excited; emotional resonance	“I was truly moved and shocked by the history behind the Great Wall.” (I01)	Emotional Arousal (EA)
Cultural meaning endorsement	Identity, cultural confidence, stewardship	cultural pride; protecting the Great Wall; cultural confidence; shared cultural spirit	“The experience strengthened my cultural confidence and made me feel that protecting this heritage is meaningful.” (I06)	Cultural Value Identification (CVI)
Psychological involvement	Absorption, story involvement, reduced external awareness	being absorbed; reduced awareness of surroundings; being part of the story; completely immersed	“I felt absorbed in the scene and focused on the task, almost forgetting the surroundings for a while.” (I13)	Psychological Immersion (IM)
Behavioral extension	Continuance, recommendation, offline interest	play again; recommend to friends; save/share; visit the real site	“The visual impact made me want to visit the Great Wall in person and recommend the experience to others.” (I04)	Behavioral Intention (BI)

Participant excerpts were collected in Chinese and translated into English by the authors. “S” refers to open-ended survey responses and “I” to interview transcripts. Fuller coding dictionaries, additional excerpts, and item-level mapping are provided in Supplementary file S3.

#### 3.4.3 Coding reliability

Two trained author-coders participated in the qualitative coding. To improve reporting transparency, the coding procedure documented coding units, code definitions, disagreement resolution, and supporting excerpts, following qualitative reporting recommendations (O’Brien et al., 2014; Tong et al., 2007). Before formal coding, the coders reviewed the codebook together and clarified inclusion and exclusion criteria, as well as rules for handling overlapping expressions. A coding unit was defined as a meaningful phrase, sentence, or short passage referring to one experiential perception or intended action. When one passage contained multiple meanings, coders identified the primary code and recorded relevant secondary meanings in analytic notes.

To assess coding consistency, both coders independently coded 5 of the 16 interview transcripts using the shared codebook. Intercoder agreement was assessed using Cohen’s *κ* (Cohen, 1960), and the result indicated substantial agreement (κ = 0.78; Landis and Koch, 1977). Disagreements were reviewed item by item and resolved through discussion. The codebook was then refined by clarifying ambiguous boundaries, especially among experiential value, emotional activation, cultural identification, and psychological involvement. The remaining transcripts and open-ended responses were coded using the refined codebook. Coding decisions, revised code definitions, representative excerpts, and item-level mapping were retained in Supplementary file S3 as an audit trail.

### 3.5 Measures

All constructs were measured immediately after the mobile session using five-point Likert items (1 = strongly disagree, 5 = strongly agree). Item wording was adapted from established scales and lightly rephrased to fit the short-session, task-based mobile XR context; Appendix A summarizes construct definitions and sources. Full measurement items are provided in the Supplementary Table S1.

#### 3.5.1 XR Technology Perception (XTP)

XTP was measured with nine items covering XR system/interaction cues, perceived usefulness, and perceived ease of use (Davis, 1989; Venkatesh and Bala, 2008). Although these components are conceptually distinguishable in classical information-systems and technology-acceptance research, they were used in this study to capture users’ context-specific holistic technical appraisal of the short-session mobile XR experience. This appraisal reflects whether the interaction felt smooth, controllable, understandable, useful, and easy enough to continue. The appropriateness of this operationalization was further examined through supplementary measurement-model comparisons.

#### 3.5.2 Narrative design (ND)

ND assessed the perceived coherence, guidance, and progression of the story–task flow, including the extent to which the narrative structure helped users understand what to do and how to interpret the experience. Items were developed for this program while aligning with narrative engagement, transportation, and interactive storytelling perspectives (Busselle and Bilandzic, 2009; Green and Brock, 2000; Jenkins, 2006; Ryan, 2015).

#### 3.5.3 Experiential value (4E)

Experiential value was assessed using adapted 4E items covering Entertainment, Education, Esthetics, and Escapism (Bassano et al., 2019; Mehmetoglu and Engen, 2011). These four dimensions were not treated as conceptually identical. Rather, in the present short-session mobile XR context, they were used to indicate users’ overall experiential-value appraisal of the brief heritage session. This modeling decision reflects the compressed and integrated nature of the story–task experience and was examined empirically in the measurement analyses.

#### 3.5.4 Emotional arousal (EA)

EA captured general activated affect during the session, such as feeling moved, impressed, or emotionally stimulated. The items were adapted from the Destination Emotion Scale tradition and rephrased for the mobile XR heritage context (Hosany and Gilbert, 2010). Qualitative materials also contained affective expressions such as being moved, shocked, excited, and emotionally resonant, which supported the contextual relevance of the EA construct. However, the measure was not designed to distinguish a full range of heritage-specific emotions, such as awe, nostalgia, curiosity, pride, or historical reflection.

#### 3.5.5 Cultural value identification (CVI)

CVI measured users’ recognition, endorsement, and perceived self-relevance of the cultural meaning conveyed by the experience. Items were adapted from social identification measures and contextualized to heritage-value identification (Cameron, 2004; Leach et al., 2008). This contextualization was also informed by heritage interpretation theory, which emphasizes that heritage communication should connect cultural meanings with visitors’ own experience, values, and sense of relevance rather than merely transmit factual information (Ham, 2016; Tilden, 2009). In this study, CVI refers to participants’ perceived alignment with and acknowledgment of the cultural values communicated through the Great Wall E-tour, rather than to pre-existing national identity or deep cultural belonging.

#### 3.5.6 Psychological immersion (IM)

IM assessed users’ subjective psychological involvement during the mobile XR session, including attentional absorption, felt involvement in the story–task flow, and reduced awareness of the surrounding environment. The items were adapted from established immersion- and presence-related inventories and streamlined for a brief post-session survey (Jennett et al., 2008; Slater and Wilbur, 1997; Witmer and Singer, 1998). In this study, IM was treated as a user-level psychological state rather than as objective technological immersiveness, system-level XR capability, or a direct equivalent of spatial presence, flow, or narrative transportation.

#### 3.5.7 Behavioral intention (BI)

BI captured immediate post-experience behavioral intention, including continuance intention, recommendation/sharing intention, and offline visit interest. The items drew on post-adoption and technology-use intention measures, with one heritage-tailored spillover indicator reflecting interest in offline follow-up (Bhattacherjee, 2001; Utami et al., 2022; Venkatesh et al., 2003). BI should therefore be interpreted as self-reported intention immediately after the session rather than as observed behavior.

### 3.6 Data analysis

Analyses followed a staged pipeline. We first examined the empirical structure of the 36 items using exploratory factor analysis (EFA) in SPSS 31. The analysis used principal axis factoring, oblique rotation, and listwise deletion. Common method bias was assessed in two steps. Harman’s single-factor test was used as an initial diagnostic, and an unmeasured common latent factor (CLF) model was then estimated in AMOS to examine whether a common method component dominated the measurement structure.

We then evaluated the measurement model in AMOS 31 using confirmatory factor analysis (CFA). We reported standardized loadings, internal consistency, convergent validity, and discriminant validity using the Fornell–Larcker criterion and heterotrait–monotrait (HTMT) ratios. Descriptive statistics and Pearson correlations were then computed for the main constructs. Hypotheses were tested using covariance-based structural equation modeling (CB-SEM) with maximum likelihood estimation, and indirect effects, including IM-related indirect routes, were evaluated with bias-corrected bootstrapping based on 5,000 resamples. Indirect effects were treated as significant when the 95% bias-corrected confidence interval excluded zero.

To address construct-level and model-boundary concerns, we further conducted several supplementary checks within a CB-SEM-oriented and sensitivity-analysis framework. These checks included XTP measurement-model comparisons, an additional common-method-bias assessment using the CLF model, prior-XR-experience control and interaction analyses, and alternative structural models examining reverse-ordering and parallel-mechanism specifications for EA, CVI, and IM.

## 4 Results

### 4.1 Preliminary tests: scale structure and common-method bias

We first examined the item structure using an exploratory factor analysis in SPSS 31 (principal axis factoring; oblique rotation with Kaiser normalization; listwise deletion). The data were suitable for factor analysis (KMO = 0.954; Bartlett’s test of sphericity, *p* < 0.001). A seven-factor solution (eigenvalues > 1) was retained and yielded a coherent pattern with limited cross-loadings. Table 3 summarizes the EFA pattern. The 4E items formed a common experiential-value factor, and the XR system/interaction cue items grouped together with PU/PEOU. This suggests that participants tended to treat these cues as an integrated appraisal of the mobile XR technical experience. This empirical pattern supports the context-specific use of XTP and 4E as overall appraisal constructs in the present short-session setting, while not implying that their subcomponents are conceptually identical across all contexts.

**Table 3:** Exploratory factor analysis (EFA) factor composition summary (*N* = 353).

Factor	Construct	k	Loading (mean)	h^2^ range	
				Low	High
F1	Experiential value (4E)	12	0.772	0.591	0.669
F2	XR Technology Perception (XTP)	9	0.753	0.567	0.626
F3	Behavioral Intention (BI)	3	0.742	0.577	0.591
F4	Psychological Immersion (IM)	3	0.765	0.518	0.604
F5	Cultural Value Identification (CVI)	3	0.746	0.557	0.567
F6	Narrative Design (ND)	3	0.724	0.499	0.519
F7	Emotional Arousal (EA)	3	0.699	0.526	0.560

Extraction: principal axis factoring; rotation: oblique with Kaiser normalization; listwise deletion. k = number of items. Esthetics is coded as AE to distinguish it from EA.

To gauge potential common method bias, we first ran Harman’s single-factor test using an unrotated principal components solution. The first factor explained 37.891% of the total variance, suggesting that no single factor accounted for the majority of item variance. However, because Harman’s test provides only a preliminary diagnostic, we further estimated an unmeasured common latent factor (CLF) model. The CLF model fit indices were χ^2^ = 573.126, df = 572, χ^2^/df = 1.002, *p* = 0.479, CFI = 1.000, TLI = 1.000, NFI = 0.932, RMSEA = 0.002, and AIC = 761.126. After adding the CLF, the substantive standardized factor loadings remained acceptable, ranging from 0.621 to 0.735, while standardized CLF loadings ranged from 0.376 to 0.422. These values indicate that a moderate, non-negligible common method component was present. Thus, although the substantive measurement structure remained interpretable, common method variance may have inflated some associations among the self-report measures and cannot be ruled out. The cross-sectional self-report findings should therefore be interpreted with appropriate caution.

### 4.2 Measurement model assessment

A confirmatory factor analysis indicated that the measurement model fit the data adequately (Table 4). Standardized loadings were statistically significant, and reliability and convergent validity indices were within commonly used benchmarks (Table 5). Discriminant validity was assessed using the Fornell–Larcker criterion (Supplementary Table S4) and cross-checked with HTMT ratios (Supplementary Table S5); both checks were consistent with construct distinctiveness. The CFA specification was kept theory-driven, without adding correlated residuals based on modification indices and without item parcelling.

**Table 4:** Model fit indices for the measurement and structural models.

Model	χ^2^	df	χ^2^/df	CFI	NFI	RMSEA	SRMR
CFA (measurement model)	583.445	574	1.016	0.999	0.931	0.007	0.028
SEM (structural model)	617.024	580	1.064	0.995	0.927	0.013	0.039

AMOS 31 with maximum likelihood estimation. The CFA model was specified a priori, without post-hoc correlated residuals or item parcelling. The SEM model corresponds to Model 2.

**Table 5:** Reliability and convergent validity.

Construct	Std.	Cronbach’s α	CR	AVE
XTP	0.753–0.792	0.932	0.928	0.604
4E	0.766–0.820	0.953	0.939	0.626
ND	0.755–0.780	0.815	0.825	0.596
IM	0.758–0.834	0.839	0.841	0.638
CVI	0.779–0.815	0.840	0.841	0.638
EA	0.771–0.807	0.827	0.827	0.615
BI	0.804–0.823	0.854	0.854	0.662

Std. = standardized CFA loadings; CR = composite reliability; AVE = average variance extracted.

### 4.3 Descriptive statistics and correlations

Descriptive statistics and correlations are reported in Table 6. Correlations were generally positive and largely statistically significant, while their magnitudes did not indicate extreme overlap among constructs.

**Table 6:** Descriptive statistics and correlations among study variables (*N* = 353).

Variable	Mean	SD	1	2	3	4	5	6	7
1. XTP	29.762	8.411	1						
2. 4E	40.785	11.422	0.407**	1					
3. ND	9.980	2.961	0.427**	0.405**	1				
4. EA	9.915	3.149	0.451**	0.450**	0.396**	1			
5. CVI	10.054	3.149	0.442**	0.430**	0.447**	0.436**	1		
6. IM	10.014	3.008	0.377**	0.414**	0.377**	0.462**	0.404**	1	
7. BI	9.779	3.309	0.476**	0.432**	0.471**	0.491**	0.440**	0.432**	1

Pearson correlations are reported. Composite scores were summed across items for each construct. **p < 0.01.

Robustness and sensitivity were examined through CB-SEM-oriented and supplementary checks reported in Sections 4.1 and 4.5. These checks included the XTP measurement-model comparison, common-method-bias diagnostics, prior-XR-experience analyses, and alternative structural models for the ordering of EA, CVI, and IM.

### 4.4 Structural model and mediation tests

**Figure 4:**
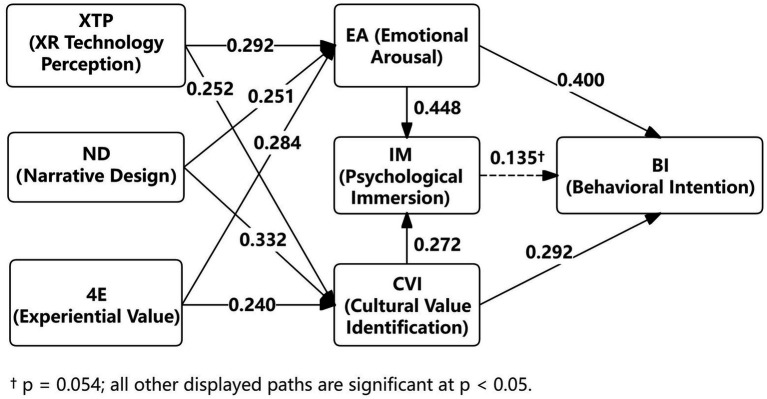
Standardized structural model results.

We then estimated the hypothesized structural model, reported as Model 2 in the model-comparison table. The model showed good global fit (Table 4). Figure 4 presents the standardized direct effects, and the full path estimates are reported in Table 7. XTP, ND, and 4E were all positively associated with both EA and CVI, supporting H1a–H3b. In the theory-specified model, EA and CVI were also positively associated with IM, consistent with H4a and H4b. The EA → IM association was stronger than the CVI → IM association.

**Table 7:** Hypothesis testing results.

Hypothesis	Path	Estimate	S. E.	C. R.	p	Std. β	Result
H1a	XTP → EA	0.329	0.071	4.634	< 0.001	0.292	Supported
H1b	XTP → CVI	0.287	0.071	4.057	< 0.001	0.252	Supported
H2a	ND → EA	0.273	0.074	3.679	< 0.001	0.251	Supported
H2b	ND → CVI	0.367	0.077	4.788	< 0.001	0.332	Supported
H3a	4E → EA	0.295	0.063	4.688	< 0.001	0.284	Supported
H3b	4E → CVI	0.252	0.063	4.018	< 0.001	0.239	Supported
H4a	EA → IM	0.422	0.064	6.556	< 0.001	0.448	Supported
H4b	CVI → IM	0.253	0.060	4.245	< 0.001	0.272	Supported
H5	IM → BI	0.149	0.077	1.930	0.054	0.135	Not supported (marginal)
H6a	EA → BI	0.418	0.076	5.499	< 0.001	0.400	Supported
H6b	CVI → BI	0.301	0.066	4.579	< 0.001	0.292	Supported

Estimate = unstandardized regression coefficient; S. E. = standard error; C. R. = critical ratio; Std. β = standardized regression coefficient. Marginal: 0.05 ≤ *p* < 0.10.

At the outcome stage, EA and CVI showed statistically significant direct associations with immediate post-experience BI, supporting H6a and H6b. The IM → BI path was positive but marginal rather than conventionally significant (Estimate = 0.149, S. E. = 0.077, C. R. = 1.930, *p* = 0.054, *β* = 0.135; Table 7). Therefore, H5 was not supported at the conventional *p* < 0.05 level. This near-threshold positive result suggests that IM should not be interpreted as irrelevant. Rather, its direct role in immediate BI appears weaker and more context-sensitive than the direct routes through EA and CVI. This pattern is plausible because a 10–20 min interruptible smartphone session may activate emotional and value-based responses quickly, but may not allow psychological immersion to stabilize into a durable state that directly translates into immediate intention.

Indirect effects were then examined using bias-corrected bootstrapping with 5,000 resamples. As shown in Table 8, the single-step indirect paths through EA and CVI were consistently supported across the three antecedent domains. By contrast, IM-related indirect routes were smaller in magnitude and less stable. This suggests that IM functioned more as a related involvement state than as a necessary pathway in the theory-specified model. These indirect effects should be interpreted as theoretically specified associations rather than as evidence of fixed temporal causality.

**Table 8:** Bootstrap indirect effects on behavioral intention (BI).

Indirect path	Estimate	95% CI	
		Low	High
XTP → EA → BI	0.138	0.067	0.239
XTP → CVI → BI	0.086	0.036	0.159
XTP → EA → IM → BI	0.021	0.001	0.053
XTP → CVI → IM → BI	0.011	0.001	0.036
ND → EA → BI	0.114	0.049	0.216
ND → CVI → BI	0.110	0.050	0.199
ND → EA → IM → BI	0.017	0.001	0.046
ND → CVI → IM → BI	0.014	0.001	0.041
4E → EA → BI	0.123	0.061	0.205
4E → CVI → BI	0.076	0.031	0.140
4E → EA → IM → BI	0.019	0.001	0.050
4E → CVI → IM → BI	0.009	0.001	0.029
EA → IM → BI	0.063	−0.002	0.143
CVI → IM → BI	0.038	0.001	0.101

Unstandardized indirect effects are reported with bias-corrected 95% bootstrap confidence intervals based on 5,000 resamples.

### 4.5 Competing models and robustness

To examine the robustness, parsimony, and interpretive boundaries of the proposed mechanism structure, we reorganized the supplementary analyses within a CB-SEM-oriented framework. The initial model comparisons were estimated using maximum likelihood and evaluated through global fit indices, information criteria, and χ^2^ difference tests (Table 9). Relative to the constrained baseline model, Model 1, which specified dual mediation through EA and CVI, showed a substantial improvement in fit (Δχ^2^ = 287.898, Δdf = 5). Model 2 then added the theoretically motivated ND → CVI and IM → BI paths and further improved fit (Δχ^2^ = 67.820, Δdf = 2). Because the IM → BI path in Model 2 was close to, but did not reach, the conventional significance threshold, we also estimated a trimmed variant, Model 2′, with the IM → BI path removed. Fit did not significantly deteriorate relative to Model 2 (Δχ^2^ = 3.663, Δdf = 1), suggesting that the direct IM → BI link is positive but model-sensitive in the present short-session context.

**Table 9:** Competing and alternative structural model comparisons.

Fit index	Model 0	Model 1	Model 2	Model 2′	Model 3	Alternative A	Alternative B
NPAR	79	84	86	85	89	82	90
df	587	582	580	581	577	584	576
χ^2^ (CMIN)	972.742	684.844	617.024	620.687	592.995	765.714	600.239
χ^2^/df	1.657	1.177	1.064	1.068	1.028	1.311	1.042
CFI	0.951	0.987	0.995	0.995	0.998	0.956	0.997
NFI	0.885	0.919	0.927	0.928	0.930	0.839	0.929
RMSEA (90% CI)	0.043 (0.038–0.048)	0.022 (0.014–0.029)	0.013 (0.000–0.022)	0.014 (0.000–0.023)	0.009 (0.000–0.020)	0.042 (0.033–0.050)	0.011 (0.000–0.021)
SRMR	0.179	0.072	0.039	0.055	0.035	0.124	0.051
AIC	1130.742	852.844	789.024	790.687	770.995	929.714	780.239
BIC	1436.193	1177.627	1121.540	1119.337	1115.111	1191.536	1128.221
Δχ^2^	—	287.898	67.820	3.663	24.029	—	—
Δdf	—	5	2	1	3	—	—
Nested reference	—	vs Model 0	vs Model 1	vs Model 2	vs Model 2	Non-nested alternative	Non-nested alternative

AIC/BIC: smaller values indicate better relative fit. Δχ^2^ tests were applied only to nested model comparisons; Alternatives A and B were non-nested specifications.

Model 3 further added direct paths from the upstream antecedents to BI. This expanded model showed slightly better global fit and lower AIC/BIC than Model 2. We treated it as a sensitivity specification rather than the primary model because it relaxes the theory-specified mediation structure and adds paths that were not central to the hypothesized mechanism. Model 2 was therefore retained as the main hypothesis-testing model, as it provides a more parsimonious and theoretically aligned representation of the proposed EA/CVI–IM mechanism while still showing excellent fit.

Additional sensitivity checks were conducted to address construct-level and model-boundary concerns. For XTP, a one-factor model showed excellent fit (χ^2^ = 23.261, df = 27, χ^2^/df = 0.862, *p* = 0.671, CFI = 1.000, TLI = 1.003, NFI = 0.988, RMSEA = 0.000, AIC = 59.261). A three correlated-factor model separating XR system/interaction cues, perceived usefulness, and perceived ease of use also showed excellent fit (χ^2^ = 19.136, df = 24, χ^2^/df = 0.797, *p* = 0.745, CFI = 1.000, TLI = 1.004, NFI = 0.991, RMSEA = 0.000, AIC = 61.136). Although the three-factor model confirmed that the components are empirically distinguishable, the one-factor model showed a slightly lower AIC and a non-significant χ^2^ difference relative to the three-factor model (Δχ^2^ = 4.125, Δdf = 3, *p* = 0.248). This supports the use of XTP as a parsimonious, context-specific holistic technical appraisal in the present mobile XR setting.

Prior XR experience was also examined as a possible boundary condition. When prior XR experience was entered as a control variable, the core XTP → EA and XTP → CVI paths remained significant (*β* = 0.293 and β = 0.252, respectively, both *p* < 0.001). Supplementary interaction tests using composite scores further showed that XTP × XR_GROUP did not significantly predict EA (B = 0.0333, *p* = 0.740) or CVI (B = −0.0838, *p* = 0.401). These results suggest that the core XTP-related psychological pathways were not simply attributable to prior XR experience and were not significantly moderated by frequent versus non-frequent XR use.

Finally, we tested alternative structural specifications for the ordering among EA, CVI, and IM. The reverse-ordering model, in which IM preceded EA and CVI (Alternative A), showed weaker fit than Model 2 (χ^2^ = 765.714, df = 584, χ^2^/df = 1.311, CFI = 0.956, NFI = 0.839, RMSEA = 0.042 [90% CI: 0.033–0.050], SRMR = 0.124, AIC = 929.714). The parallel-mechanism model, in which EA, CVI, and IM jointly predicted BI (Alternative B), showed excellent fit (χ^2^ = 600.239, df = 576, χ^2^/df = 1.042, CFI = 0.997, NFI = 0.929, RMSEA = 0.011 [90% CI: 0.000–0.021], SRMR = 0.051, AIC = 780.239). Compared with Model 2, Alternative B showed slightly better χ^2^/df, CFI, RMSEA, and AIC, but higher BIC and SRMR. Because Alternatives A and B are not nested in Model 2, χ^2^ difference tests were not applied.

Accordingly, Model 2 was retained as the theory-specified hypothesis model rather than as a purely data-driven best-fitting model. This decision was made because Model 2 follows the proposed theoretical framework and provides a parsimonious basis for testing the hypothesized EA/CVI–IM mechanism. Alternative B nevertheless provides important evidence that EA, CVI, and IM may also operate as closely related or parallel psychological mechanisms in short-session mobile XR experiences. The EA/CVI → IM pathway is therefore interpreted cautiously as a theoretically specified association rather than a definitive temporal sequence.

## 5 Discussion

### 5.1 Key findings and mechanism interpretation

This study examined how immediate post-experience behavioral intention is formed in short-session mobile XR cultural-heritage storytelling. The results suggest that BI is more closely associated with a cluster of psychological mechanisms than with immersion alone. Across the three antecedent domains—XR Technology Perception (XTP), Narrative Design (ND), and Experiential Value (4E)—each was positively associated with Emotional Arousal (EA) and Cultural Value Identification (CVI). In the theory-specified model, EA and CVI were also positively associated with Psychological Immersion (IM), and both showed stable direct associations with BI. The direct IM → BI path was positive but marginal in the main model, while IM became significant in the supplementary parallel-mechanism model. This pattern suggests that affective activation, cultural value identification, and immersion are closely related mechanisms in short-session mobile XR experiences, although their temporal ordering cannot be treated as definitively established by the present cross-sectional data.

A further nuance emerged from the mediation and alternative-model results. Indirect effects from XTP, ND, and 4E to BI were mainly carried through single-step routes via EA and CVI, whereas IM-related indirect routes were smaller and more model-sensitive. The supplementary parallel model further indicated that EA, CVI, and IM may also operate simultaneously in shaping BI. Thus, immersion should not be dismissed as unimportant in short sessions, but neither should it be assumed to function as the necessary pathway. Rather, IM appears to be an involvement-related state whose behavioral role may depend on model specification, session length, and the extent to which the experience allows sustained attention to stabilize.

### 5.2 Dialogue with prior research

The findings are broadly consistent with technology-to-behavior accounts. TAM and the information-systems success and user-evaluation literature suggest that usefulness, ease of use, and system-quality cues shape downstream user responses (Davis, 1989; DeLone and McLean, 2003; Venkatesh and Bala, 2008). This study extends that logic to mobile heritage storytelling. Technical appraisal (XTP) was associated not only with acceptance-oriented responses, but also with affective activation and value-based identification. In short-session XR interaction, responsiveness, controllability, and clear feedback may function as experience-shaping cues that support immediate emotional activation and meaning processing, consistent with presence and immersion perspectives that link perceived control and realism to subjective experience quality (Lessiter et al., 2001; Slater and Wilbur, 1997; Witmer and Singer, 1998).

The results for ND also align with interactive storytelling and narrative engagement perspectives. ND was positively associated with both EA and CVI, suggesting that coherent progression, guided pacing, and story–task coupling can intensify affective response while scaffolding interpretation (Green and Brock, 2000; Jenkins, 2006; Murray, 2017; Ryan, 2015). Its comparatively stronger association with CVI is especially plausible in heritage contexts, where role framing and interpretive structure can help move cultural information from simple exposure toward personally endorsed meaning.

Experiential Value (4E) likewise functioned as an upstream organizer of experience cues. Rather than operating only as a post-hoc evaluative frame, overall experiential value was positively associated with EA and CVI, supporting the view that rapid experiential appraisal contributes to both affective activation and value-based identification during use (Bassano et al., 2019; Mehmetoglu and Engen, 2011; Oh et al., 2007). In the theory-specified model, EA and CVI were associated with IM, which is consistent with the view of immersion as an involvement state supported by motivational energy and interpretive commitment. At the same time, the alternative-model checks indicate that these psychological mechanisms may also operate in parallel, particularly in compressed mobile sessions.

### 5.3 Theoretical contributions

This study contributes to media-psychological research on mobile XR heritage storytelling by reframing immersion as one part of a broader mechanism pattern involving affective activation, cultural value identification, and psychological involvement. The findings show that XTP, ND, and 4E are not only design or experience attributes, but also antecedent conditions linked to users’ immediate emotional and meaning-based responses.

A further contribution lies in clarifying the role of IM within the present short-session mobile XR context. In this setting, EA and CVI showed the most stable direct associations with BI, whereas IM appeared as a related involvement state whose role was more sensitive to model specification. This does not mean that immersion is unimportant. Rather, it suggests that in brief and interruptible mobile XR sessions, affective and value-based responses may connect more directly with immediate intention, while immersion may depend on whether the session allows sustained involvement to develop.

By integrating TAM-based technical appraisal, experience-economy value cues, and narrative-task structure within a single model, the study offers an integrative account of how technical entry conditions, story guidance, and experiential cues jointly shape emotion, cultural meaning, and involvement in mobile heritage communication. The contribution is not to prove a fixed causal order among EA, CVI, and IM, but to identify a theoretically grounded mechanism pattern that can guide future research on short-session XR heritage experiences.

### 5.4 Design and practical implications

The findings suggest that behavior-oriented XR heritage design should begin with a clear and low-friction story–task structure. Visual and spatial presentation, interaction affordances, and feedback loops are likely to matter most when embedded in role identity and plot progression. Examples include guardian roles, restoration tasks, guided choices, and stepwise missions. In short sessions, users have little time to infer what to do next; a coherent next-action structure can help technical interaction become affectively and culturally meaningful.

Responsiveness and feedback should also be treated as time-sensitive design cues. Rapid feedback, micro-achievements, badges, collectible artifacts, or progress visualization may be most effective when they appear at moments of narrative reveal or task completion. These moments can briefly heighten action readiness, so they should be paired with low-friction gateways such as continue, save, share, recommend, or offline follow-up prompts.

CVI is more likely to emerge when cultural meaning is built through coordinated cues rather than direct persuasion. Educational prompts should be embedded in tasks and dialogue, while visual atmosphere, role-based participation, and scene progression should support credible cultural interpretation. This helps users move from receiving heritage information to recognizing and endorsing cultural value.

Immersion should be designed as an experience-deepening condition rather than assumed to convert intention on its own. A feasible design strategy is to secure EA and CVI early, use continuity cues and coherent pacing to sustain involvement, and provide meaningful closure through a personalized recap, commemorative artifact, next-episode cue, or in-person route suggestion. In this sense, immersion remains valuable, but it works best when connected to clear action bridges.

### 5.5 Limitations and future research

Several limitations should be considered. BI was assessed as immediate post-session intention rather than observed behavior. The findings therefore speak to intention-formation mechanisms, not actual behavioral outcomes. All constructs were also measured through a cross-sectional self-report survey immediately after the session. The results should therefore be interpreted as associations among perceived experience variables rather than as evidence of strict causal effects. Although the CLF analysis indicated that the substantive measurement structure remained interpretable, it also suggested a moderate common method component. Method-related inflation of some associations cannot be ruled out.

The participant profile also limits generalizability. All participants were Chinese and recruited in China, and the sample was relatively homogeneous in educational background and mobile-use familiarity. This helped reduce noise from basic operational barriers, but it may also have contributed to the clean factor structure and high model fit. The findings should therefore be understood as evidence from a Chinese, university-based, mobile-proficient participant sample, rather than as direct evidence for all heritage-tourism populations or international audiences.

The measures also have boundaries. EA captured general activated affect rather than a full heritage-specific emotional profile. Although the qualitative materials included expressions such as being moved, shocked, excited, and emotionally resonant, the quantitative scale did not distinguish discrete heritage-related emotions. Future studies should examine whether emotions such as awe, nostalgia, curiosity, pride, historical reflection, or moral elevation relate differently to CVI, IM, and BI. Similarly, the overall 4E operationalization fits the present brief mobile session. Longer and more differentiated heritage experiences, especially HMD-based XR scenarios with extended engagement, should compare this overall 4E treatment with dimension-specific or higher-order specifications. Although discriminant validity was supported statistically, some items related to XR realism, escapism, and immersion shared adjacent experiential language. Future studies should use more differentiated item wording or experimental manipulation to distinguish perceived technical realism, escapist value, and psychological immersion more clearly. The cross-sectional post-experience survey design also limits the interpretation of directionality among EA, CVI, and IM. Although the theory-specified model positioned EA and CVI as proximal psychological responses associated with IM, the supplementary parallel-mechanism model was also statistically plausible. The present findings should therefore not be interpreted as evidence of a definitive temporal sequence.

Future research can strengthen the model in several directions. Behavioral traces and follow-up measures could be integrated, including completion, drop-off, replay, sharing clicks, artifact saving, route-link taps, and delayed checks of revisit or in-person planning. Controlled design experiments could vary narrative scaffolding, story–task coupling, or feedback timing while holding content constant, allowing more direct tests of how shifts in EA and CVI relate to BI. Broader samples and contexts are also needed, including more diverse visitor groups, international audiences, and comparisons between smartphone-based and headset-based XR. Longitudinal, experimental, and process-tracing designs would be especially useful for examining whether EA and CVI precede IM, whether IM reinforces emotional and value-based responses, or whether these mechanisms operate simultaneously.

Taken together, the study suggests that short-session mobile XR cultural-heritage storytelling links technical, narrative, and experiential cues with immediate post-experience BI through a related mechanism cluster involving EA, CVI, and IM. In the theory-specified model, effects from XTP, ND, and 4E to BI were transmitted mainly through EA and CVI, while IM showed a weaker and more model-sensitive role. For design practice, brief mobile XR heritage experiences should ensure smooth technical entry, coherent role-driven story–task progression, and early activation of emotion and cultural value, while also offering immersion-supporting continuity and clear action bridges for later engagement.

## Data Availability

The datasets presented in this study can be found in online repositories. The names of the repository/repositories and accession number(s) can be found in the article/Supplementary material.

## Appendix A: construct definitions, item counts, and sources.

**Table:**

Construct (abbr.)	Operational definition (in this study)	No. of items	Measurement and contextual source(s)
XR Technology Perception (XTP)	Holistic appraisal of mobile XR technical experience combining XR system/interaction cues and TAM constructs (PU/PEOU).	9 (XR1–XR3, PU1–PU3, PEOU1–PEOU3)	Schubert et al. (2001), DeLone and McLean (2003), Wixom and Todd (2005). Davis (1989), Venkatesh and Bala (2008).
Narrative Design (ND)	Perceived quality of narrative organization and guidance (coherence, engagement, task-driven storytelling).	3 (ND1–ND3)	Green and Brock (2000), Busselle and Bilandzic (2009).
Experiential value (4E)	Perceived experiential value across Entertainment, Education, Escapism and Esthetics.	12 (EN1–EN3, ED1–ED3, ES1–ES3, AE1–AE3)	Oh et al. (2007), Mehmetoglu and Engen (2011).
Emotional Arousal (EA)	General activated affect during the experience, such as feeling moved, impressed, or emotionally stimulated.	3 (EA1–EA3)	Hosany and Gilbert (2010).
Cultural Value Identification (CVI)	Recognition and internalization of cultural meaning/value conveyed by the narrative content (value alignment and identification).	3 (CVI1–CVI3)	Cameron (2004), Leach et al. (2008), contextual support: Tilden (2009), Ham (2016).
Psychological Immersion (IM)	Subjective immersion during the session (absorption, reduced awareness of surroundings, being part of the story).	3 (IM1–IM3)	Witmer and Singer (1998), Jennett et al. (2008).
Behavioral Intention (BI)	Continuance intention, recommendation intention, and offline visit interest.	3 (BI1–BI3)	Davis (1989), Utami et al. (2022), Bhattacherjee (2001).

Full measurement items are provided in Supplementary Table S1. AE denotes Esthetics and is used to distinguish it from EA, Emotional Arousal.
