# Supplementary material for: Modeling immersion pathways in XR-based cultural heritage IP narrative experiences: an integrated approach based on TAM, experience economy, and grounded theory
Source: Front Psychol. 2026 Jun 10;17:1818614. doi: 10.3389/fpsyg.2026.1818614 (PMC13290812; doi:10.3389/fpsyg.2026.1818614)
Supplement: Supplementary file 1 [file Data_sheet_1.zip › Data Sheet New/Text S1. IInterview guide.pdf]

Appendix A. Semi-Structured Interview Guide – XR Cultural Narrative Experience (Cloud Tour Great Wall App)

（中文–English bilingual version / 中英对照版）

No.	中文提问	English Question
Q1	您对本次“云游长城”应用的整体体验感受如何？	How would you describe your overall experience of using the “Cloud Tour Great Wall” app?
Q2	在操作过程中，是否遇到过任何不便或障碍？您认为有哪些方面可以改进？	During the interaction, did you encounter any inconvenience or obstacles? What aspects do you think could be improved?
Q3	您在使用过程中最印象深刻的环节是什么？（如某个场景、任务、角色或声音等）	What was the most memorable part of the experience (e.g., a scene, task, character, or sound)?
Q4	本次体验是否加深了您对长城这一文化遗产的理解与印象？请简要说明理由。	Did this experience deepen your understanding or impression of the Great Wall as a cultural heritage site? Please briefly explain why.
Q5	您认为这种基于 XR 技术的虚拟体验方式，是否对未来实地参观长城或类似文化遗址有激发作用？是否具有“预体验”的价值？	Do you think this XR-based virtual experience could motivate you to visit the Great Wall (or similar heritage sites) in person in the future? Do you perceive it as having “pre-visit” value?

Interviewer note: Use follow-up prompts when needed (e.g., “Could you give an example?”, “What made you feel that way?”, “At what moment did it occur?”).

访谈员提示：可根据回答进行追问（如“能举个例子吗？”“为什么会这样感觉？”“是在体验的哪个环节发生的？”）。

Appendix B. Interview Notes Template (One page per participant)

（中文–English bilingual version / 中英对照版）

Participant ID / 受访编号: __________ **Gender / 性别:** __________ **Age / 年龄:** __________

Interview Date / 访谈日期: __________ **Location / 地点:** __________

Device / 使用设备: XR headset / XR 眼镜 ☐ Mobile phone / 手机 ☐ Other / 其他: ________

Q1 (中文): 您对本次"云游长城"应用的整体体验感受如何？

Q1 (EN): How would you describe your overall experience of using the "Cloud Tour Great Wall" app?

Response / 回答：

____

____

____

Q2 (中文): 在操作过程中，是否遇到过任何不便或障碍？您认为有哪些方面可以改进？

Q2 (EN): During the interaction, did you encounter any inconvenience or obstacles? What aspects do you think could be improved?

Response / 回答：

____

____

____

Q3 (中文): 您在使用过程中最印象深刻的环节是什么？（如某个场景、任务、角色或声音等）

Q3 (EN): What was the most memorable part of the experience (e.g., a scene, task, character, or sound)?

Response / 回答：

____

____

____

Q4 (中文): 本次体验是否加深了您对长城这一文化遗产的理解与印象？请简要说明理由。

Q4 (EN): Did this experience deepen your understanding or impression of the Great Wall as a cultural heritage site? Please briefly explain why.

Response / 回答：

____

____

____

Q5 (中文): 您认为这种基于 XR 技术的虚拟体验方式，是否对未来实地参观长城或类似文化遗址有激发作用？是否具有"预体验"的价值？

Q5 (EN): Do you think this XR-based virtual experience could motivate you to visit the Great Wall (or similar heritage sites) in person in the future? Do you perceive it as having "pre-visit" value?

Response / 回答：

Additional notes / 其他补充：
