# Supplementary material for: Modeling immersion pathways in XR-based cultural heritage IP narrative experiences: an integrated approach based on TAM, experience economy, and grounded theory
Source: Front Psychol. 2026 Jun 10;17:1818614. doi: 10.3389/fpsyg.2026.1818614 (PMC13290812; doi:10.3389/fpsyg.2026.1818614)

Supplementary Material

1 Supplementary Figures and Tables

Supplementary Table S1. Measurement items
Supplementary Table S2. Chi-square difference tests for nested structural model comparisons
Supplementary Table S3. Key structural path comparison between Model 2 and Model 3
Supplementary Table S4. Discriminant validity (Fornell–Larcker)
Supplementary Table S5. Discriminant validity (HTMT ratios)
Supplementary Figures S1. Model 2 CB-SEM results (AMOS 31): standardized path coefficients
Supplementary Figures S2. PLS-SEM robustness results (SmartPLS 4): standardized path coefficients

Supplementary Text S3. Grounded-theory category-to-SEM mapping details

Table S1. Measurement items

Construct	Item Code	Items	Source(s)
XTP-XR	XR1	The visual, audio, and interactive feedback of the XR system felt highly realistic.	Schubert et al. (2001); DeLone & McLean (2003); Wixom & Todd (2005)
	XR2	The system ran smoothly, and the task interactions responded in a timely way.	
	XR3	The XR system's technical performance and interaction feedback enhanced my sense of "being there" in the scene.	
XTP-PU	PU1	This app enhanced my understanding of Great Wall culture.	Davis (1989); Venkatesh & Bala (2008)
	PU2	Using this app made it easier for me to access cultural knowledge.	
	PU3	This experience was helpful for my cultural learning.	
XTP-PEOU	PEOU1	The app interface was simple and easy to operate.	
	PEOU2	I was able to complete the tasks without help from others.	
	PEOU3	Using this app was effortless for me.	
ND	ND1	The storyline and task interaction process kept me interested in exploring further.	Green & Brock (2000); Busselle & Bilandzic (2009)
	ND2	The IP characters and interactive tasks in the scenes helped me better understand the cultural content.	
	ND3	The overall narrative design was logical, culturally engaging, and made me feel involved.	
4E-Entertainment	EN1	The overall experience was fun and enjoyable.	
	EN2	I felt relaxed and entertained during the experience.	
	EN3	The content was entertaining and kept me engaged.	
4E-	ED1	I learned new cultural knowledge through this experience.	

Education	ED2	The content encouraged me to explore the related culture further.	Oh et al. (2007); Mehmetoglu & Engen (2011)
	ED3	I clearly understood the historical or cultural information conveyed in the experience.	
4E-Esthetics	AE1	The visual style and layout of the XR scenes were aesthetically pleasing.	
	AE2	I was captivated by the beauty of the experience environment.	
	AE3	The details and design style of the XR environment enhanced my overall experience.	
4E-Escapism	ES1	I completely entered a different world during the experience.	
	ES2	I felt present and actively involved during the experience.	
	ES3	This experience helped me temporarily forget real-world concerns.	
EA	EA1	I felt deeply moved or impressed during the experience.	Hosany & Gilbert (2010)
	EA2	I experienced emotional activation during the experience.	
	EA3	The XR experience evoked emotional resonance with the culture content.	
CVI	CVI1	The values conveyed through the Great Wall story felt consistent with who I am.	Cameron (2004); Leach et al. (2008); contextual support: Tilden (2009); Ham (2016).
	CVI2	I encountered cultural meanings in the app that felt personally relevant to me.	
	CVI3	The cultural spirit portrayed in the Great Wall narrative made me feel a sense of identification.	
IM	IM1	I was fully immersed in this XR experience.	Witmer & Singer (1998); Jennett et al. (2008)
	IM2	I lost awareness of the real world around me during the experience.	
	IM3	I felt like I was part of the story throughout the experience.	
BI	BI1	I intend to continue using this XR cultural experience app.	Bhattacherjee (2001); Utami et al. (2022)
	BI2	I would recommend this cultural app to others.	
	BI3	This experience made me interested in visiting the real Great Wall offline.	

Table S2. Chi-square difference tests for nested structural model comparisons

Comparison	$\Delta\chi^2$	Δdf	Result
Model 0 → Model 1	287.898	5	$p < 0.001$
Model 1 → Model 2	67.820	2	$p < 0.001$
Model 2 → Model 3	24.029	3	$p < 0.001$
Model 2 → Model 2′(remove IM → BI)	3.663	1	$p = 0.056$(marginal)

Note. Model 3 refers to the direct-effects expanded model. Alternative A and Alternative B are non-nested supplementary specifications and are therefore not included in the χ^2 difference tests. Marginal = $0.05 \le p < 0.10$; ns = $p \ge 0.10$.

Table S3. Key structural path comparison between Model 2 and Model 3

Path (→ BI)	Model 2 (Std. β, p)	Model3 (Std. β, p)
IM → BI	0.135, p = 0.054 (marginal)	0.113, p = 0.093(marginal)
EA → BI	0.400, p < 0.001	0.230, p = 0.004
CVI → BI	0.292, p < 0.001	0.111, p = 0.129(ns)
XTP → BI	—	0.170, p = 0.007
ND → BI	—	0.221, p = 0.002
4E → BI	—	0.083, p = 0.172(ns)

Note. Model 3 adds direct paths from XTP, ND, and 4E to BI and is treated as a sensitivity specification rather than the primary hypothesis-testing model. Marginal = 0.05 ≤ p < 0.10; ns = p ≥ 0.10.

Table S4. Discriminant validity (Fornell–Larcker)

Construct	XTP	ND	4E	EA	CVI	IM	BI
**XTP**	0.777						
**ND**	0.487	0.772					
**4E**	0.434	0.458	0.791				
**EA**	0.509	0.483	0.500	0.784			
**CVI**	0.500	0.539	0.486	0.520	0.798		
**IM**	0.427	0.457	0.456	0.556	0.472	0.799	
**BI**	0.534	0.563	0.477	0.583	0.521	0.503	0.814

Note. Diagonal elements are the square roots of AVE (√AVE) computed from CFA standardized loadings; off-diagonal elements are latent inter-construct correlations. Discriminant validity is supported when each √AVE exceeds the correlations between the corresponding construct and all other constructs.

Table S5. Discriminant validity (HTMT ratios)

Construct	XTP	ND	4E	EA	CVI	IM	BI
**XTP**	—						
**ND**	0.488	—					
**4E**	0.435	0.456	—				
**EA**	0.513	0.482	0.503	—			
**CVI**	0.500	0.541	0.486	0.522	—		
**IM**	0.426	0.456	0.461	0.554	0.481	—	
**BI**	0.533	0.564	0.476	0.583	0.520	0.509	—

Note. HTMT = heterotrait–monotrait ratio. All HTMT values were below the recommended threshold of 0.85, supporting discriminant validity. Bootstrapped HTMT confidence intervals based on 5,000 resamples did not include 1.00 for any construct pair.

Figures S1. Model 2 CB-SEM results (AMOS 31): standardized path coefficients.

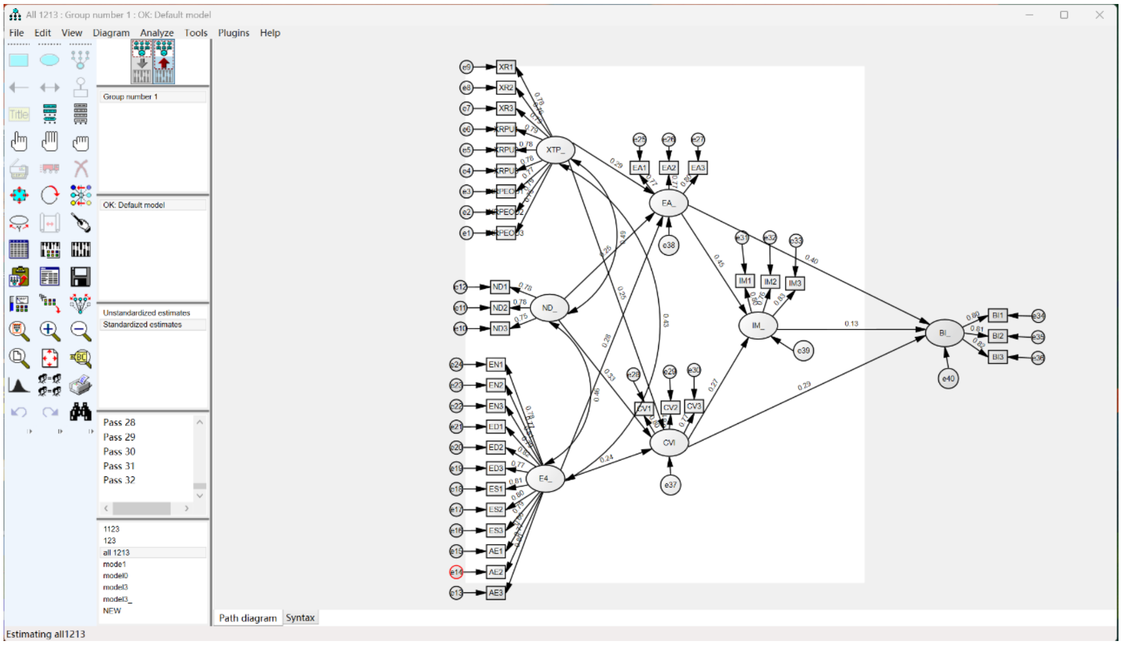

Supplement: Supplementary file 1 [file Data_sheet_1.zip › Data Sheet New/Supplementary_Material.pdf]
