# Supplementary material for: Modeling immersion pathways in XR-based cultural heritage IP narrative experiences: an integrated approach based on TAM, experience economy, and grounded theory
Source: Front Psychol. 2026 Jun 10;17:1818614. doi: 10.3389/fpsyg.2026.1818614 (PMC13290812; doi:10.3389/fpsyg.2026.1818614)
Supplement: Supplementary file 1 [file Data_sheet_1.zip › Data Sheet New/Text S3. Participant information sheet and consent form..pdf]

Text S3. Participant Information Sheet and Consent Form

A. XR On-site Experience and Interview (Qualitative Exploration)

Participant Information Sheet

Study title:

Modeling Immersion Pathways in XR Cultural Heritage IP Narrative Experiences: An Integrated Approach Based on TAM, Experience Economy Theory, and Grounded Theory

Researcher Information

Role	Name	Affiliation	Position	Contact
Principal Investigator	Dan Liu	Interdisciplinary Program of Arts & Design Technology, Chonnam National University	Doctoral student	010-2191-1632<br>13941560078
Co-researcher (Supervisor)	Jung-ho Jung	Department of Design, Chonnam National University	Professor	010-7644-4890

You are considered suitable to participate in this study and are therefore invited to take part. Before deciding whether to participate, please read the following information carefully. If you have any questions, you may ask the researchers at any time. Participation in this study is entirely voluntary, and it is important that you fully understand all information related to the study.

After you are satisfied that all of your questions have been answered, please sign the consent form. The researcher will also sign and date the document and provide you with a copy.

1. Purpose of the study

This study aims to examine what kinds of immersive experiences users may have when engaging with XR (extended reality) cultural heritage content via mobile devices, such as the Cloud Great Wall app, and to further analyse how such immersion may be transformed into behavioural intentions.

This study is informed by the following theoretical models:

- Technology Acceptance Model (TAM): explains how people accept new technologies;
- Experience Economy Theory: analyses how different types of experiences affect individuals;
- Immersion theory and grounded theory: explore how cognition and emotion influence users' sense of immersion.

At present, cultural heritage content is no longer limited to information display, but is increasingly developing into immersive digital experiences supported by XR technologies. In this process, transforming cultural knowledge and story-based content into cultural IP has become an important way to enhance enjoyment and immersion. However, existing studies have often focused on cognitive factors such as perceived usefulness (PU) and perceived ease of use (PEOU), or have simply listed experiential elements, while paying insufficient attention to the integrated relationship among emotion, cognition, immersion and behaviour.

This study therefore systematically examines how users' emotional and cognitive evaluations during the experience may elicit immersion and ultimately influence subsequent behaviours, such as whether they intend to reuse the app, recommend it to others, or visit the cultural heritage site in person.

2. Procedures to be undertaken by participants

The research procedure used in this study is an immersive cultural heritage IP experience supported by mobile XR technology. Participants will take part on site in an immersive content experience of approximately 10 to 20 minutes using the Cloud Great Wall XR application. After the experience, the following procedures will be conducted in sequence:

- Open-ended paper questionnaire: Participants will provide written descriptions of their experience during use, focusing on system usability, immersion, emotional responses and overall evaluation.
- Semi-structured in-depth interview (approximately 10 participants): After completing the questionnaire, approximately 10 participants will be selected for one-to-one interviews lasting approximately 15 minutes. The interviews will explore their immersive experience, emotional changes and user perceptions in greater depth.

Participants may choose to take part in only one of these procedures, or may complete both the questionnaire and the in-depth interview. Once the number of interview participants reaches 10, subsequent participants will only be asked to complete the open-ended questionnaire.

3. No randomisation or control group

This is a single-group descriptive study. It does not involve an experimental group or a control group, and no random assignment will be conducted.

4. What participants are asked to do

Participants are asked to remain attentive during the experience, complete the immersive procedure, and answer the paper questionnaire honestly after the experience. Some participants may also take part in a follow-up interview guided by the researcher.

5. Expected benefits

Participants may enhance their cultural understanding and digital immersive experience by engaging with XR cultural heritage content. This may contribute to individual cultural literacy and may also provide a reference for the future development of cultural tourism services. However, this study does not provide direct financial benefits.

6. Possible risks and discomfort

Using XR content may cause some participants to experience visual fatigue, mild dizziness, or emotional fluctuations related to cultural content, such as sadness or nostalgia. Such reactions are expected to be temporary and may vary from person to person. The questionnaire and interview do not involve personally identifiable information, and participants may stop participation at any time if they feel uncomfortable.

7. Response and support in case of discomfort

This study is not expected to cause physical harm. If psychological discomfort or other problems occur during participation, the research team will provide necessary psychological support and assist the participant in contacting relevant institutions or medical services. Contact: +8613941560078.

8. Compensation and reimbursement

After completing the XR experience, participants who take part in either the open-ended questionnaire or the in-depth interview will receive one souvenir worth approximately RMB 25 on site. Participants who take part in both the questionnaire and the interview may receive two identical souvenirs, up to a maximum of two. In addition, all participants will receive a one-time transportation allowance of RMB 16. All compensation and reimbursement will be provided directly by the researchers on site, and no online transfer will be made.

9. Personal expenses

Participants will not be required to pay any additional costs for this study. The space and equipment needed for the experience and questionnaire will be provided free of charge. Apart from the transportation allowance described above, any other personal expenses will be borne by the participants themselves.

10. Alternative options

Even if an individual does not participate in this study, they may still access similar XR cultural heritage content through commercially available apps or exhibition venues. Non-participation will not affect their personal learning or travel arrangements.

11. Voluntary participation and withdrawal

You may decide whether to participate after fully understanding the content of this study. You may also withdraw from participation at any time without giving a reason and without any disadvantage.

12. Protection of personal information and review authority

Your identity information will be kept strictly confidential and will not be disclosed publicly. Only the Chonnam National University Institutional Review Board may review the data for the purpose of evaluating the appropriateness and reliability of the study. All information will remain strictly confidential.

13. Notification of new information

If any new information arises during the study that may affect your judgement or safety, we will inform you promptly and ask whether you wish to continue participating.

14. Contact information for the review committee

This study has been approved by the Chonnam National University Institutional Review Board. If you have any questions about your rights or participation in the study, please contact:

Chonnam National University Institutional Review Board Tel: 062-530-5932 Email: irb@jnu.ac.kr

15. Possible discontinuation of participation

You may be excluded from the study without prior notice in the following circumstances:

- repeated failure to follow the researcher's instructions;
- requesting to stop participation due to discomfort or psychological burden during the experience.

16. Study period and number of participants

This study will be conducted from the date of IRB approval until December 2025, and data collection is expected to take approximately 10 days. Each participant will spend approximately 10 to 20 minutes completing one experience and either a questionnaire or an interview.

The study plans to recruit 50 on-site experience participants, of whom approximately 10 will take part in interviews for the qualitative study.

Participant Informed Consent Form

Study title:

Modeling Immersion Pathways in XR Cultural Heritage IP Narrative Experiences: An Integrated Approach Based on TAM, Experience Economy Theory, and Grounded Theory

- I have fully understood the contents of this study through the researcher's explanation and/or written materials.
- I have been informed of the possible risks (disadvantages) and benefits (advantages) associated with participation in this study.
- I voluntarily agree to participate in this study.
- I understand that I may refuse to participate or withdraw from the study at any time without giving a reason and without any disadvantage.
- I agree that, within the scope permitted by law and ethical review regulations, the researchers may collect and process the information I provide.
- I understand that I will receive a copy of this consent form.

Audio recording / video recording / photography: ☐ Agree / ☐ Do not agree

**Participant:**	(Signature)
**Date:**	Year ______ Month ______ Day ______
**Researcher:**	(Signature)
**Date:**	Year ______ Month ______ Day ______

B. XR Questionnaire Survey (Quantitative Study)

Participant Information Sheet

Study title:

Modeling Immersion Pathways in XR Cultural Heritage IP Narrative Experiences: An Integrated Approach Based on TAM, Experience Economy Theory, and Grounded Theory

Researcher Information

Role	Name	Affiliation	Position	Contact
Principal Investigator	Dan Liu	Interdisciplinary Program of Arts & Design Technology, Chonnam National University	Doctoral student	010-2191-1632<br>13941560078
Co-researcher (Supervisor)	Jung-ho Jung	Department of Design, Chonnam National University	Professor	010-7644-4890

You are considered suitable to participate in this study and are therefore invited to take part. Before deciding whether to participate, please read the following information carefully. If you have any questions, you may ask the researchers at any time. Participation in this study is entirely voluntary, and it is important that you fully understand all information related to the study.

After you are satisfied that all of your questions have been answered, please sign the consent form. The researcher will also sign and date the document and provide you with a copy.

1. Purpose of the study

This study aims to examine what kinds of immersive experiences users may have when engaging with XR (extended reality) cultural heritage content via mobile devices, such as the Cloud Great Wall app, and to further analyse how such immersion may be transformed into behavioural intentions.

This study is informed by the following theoretical models:

- Technology Acceptance Model (TAM): explains how people accept new technologies;
- Experience Economy Theory: analyses how different types of experiences affect individuals;
- Immersion theory and grounded theory: explore how cognition and emotion influence users' sense of immersion.

At present, cultural heritage content is no longer limited to information display, but is increasingly developing into immersive digital experiences supported by XR technologies. In this process, transforming cultural knowledge and story-based content into cultural IP has become an important way to enhance enjoyment and immersion. However, existing studies have often focused on cognitive factors such as perceived usefulness (PU) and perceived ease of use (PEOU), or have simply listed experiential elements, while paying insufficient attention to the integrated relationship among emotion, cognition, immersion and behaviour.

This study therefore systematically examines how users' emotional and cognitive evaluations during the experience may elicit immersion and ultimately influence subsequent behaviours, such as whether they intend to reuse the app, recommend it to others, or visit the cultural heritage site in person.

2. Procedures to be undertaken by participants

The research procedure used in this study is an immersive cultural heritage IP experience based on mobile XR technology. Participants will take part on site in an immersive experience lasting approximately 10 to 20 minutes using the Cloud Great Wall XR application. After the experience, the subsequent research procedure will be carried out as follows:

Based on the results of the preliminary open-ended questionnaires, interview data and relevant literature analysis, key influencing factors will be identified and a structured scale will be developed. An offline structured questionnaire

survey will then be conducted, with the planned sample size specified in the study period and participant-number section below.

3. No randomisation or control group

This is a single-group descriptive study. It does not involve an experimental group or a control group, and no random assignment will be conducted.

4. What participants are asked to do

Participants are asked to remain attentive during the experience, complete the immersive procedure, and answer the paper questionnaire honestly after the experience.

5. Expected benefits

Participants may enhance their cultural understanding and digital immersive experience by engaging with XR cultural heritage content. This may contribute to individual cultural literacy and may also provide a reference for the future development of cultural tourism services. However, this study does not provide direct financial benefits.

6. Possible risks and discomfort

Using XR content may cause some participants to experience visual fatigue, mild dizziness, or emotional fluctuations related to cultural content, such as sadness or nostalgia. Such reactions are expected to be temporary and may vary from person to person. The questionnaire does not involve personally identifiable information, and participants may stop participation at any time if they feel uncomfortable.

7. Response and support in case of discomfort

This study is not expected to cause physical harm. If psychological discomfort or other problems occur during participation, the research team will provide necessary psychological support and assist the participant in contacting relevant institutions or medical services. Contact: +8613941560078.

8. Compensation and reimbursement

After completing the XR experience, participants who take part in the questionnaire survey will receive one souvenir worth approximately RMB 25 on site. In addition, all participants will receive a one-time transportation allowance of RMB 16. All compensation and reimbursement will be provided directly by the researchers on site, and no online transfer will be made.

9. Personal expenses

Participants will not be required to pay any additional costs for this study. The space and equipment needed for the experience and questionnaire will be provided free of charge. Apart from the transportation allowance described above, any other personal expenses will be borne by the participants themselves.

10. Alternative options

Even if an individual does not participate in this study, they may still access similar XR cultural heritage content through commercially available apps or exhibition venues. Non-participation will not affect their personal learning or travel arrangements.

11. Voluntary participation and withdrawal

You may decide whether to participate after fully understanding the content of this study. You may also withdraw from participation at any time without giving a reason and without any disadvantage.

12. Protection of personal information and review authority

Your identity information will be kept strictly confidential and will not be disclosed publicly. Only the Chonnam National University Institutional Review Board may review the data for the purpose of evaluating the appropriateness and reliability of the study. All information will remain strictly confidential.

13. Notification of new information

If any new information arises during the study that may affect your judgement or safety, we will inform you promptly and ask whether you wish to continue participating.

14. Contact information for the review committee

This study has been approved by the Chonnam National University Institutional Review Board. If you have any questions about your rights or participation in the study, please contact:

Chonnam National University Institutional Review Board Tel: 062-530-5932 Email: irb@jnu.ac.kr

15. Possible discontinuation of participation

You may be excluded from the study without prior notice in the following circumstances:

- repeated failure to follow the researcher's instructions;
- requesting to stop participation due to discomfort or psychological burden during the experience.

16. Study period and number of participants

This study will be conducted from the date of IRB approval until December 2025, and data collection is expected to take approximately one month. Each participant will spend approximately 10 to 20 minutes completing one experience and the questionnaire.

The study plans to collect 300 structured questionnaire responses for the quantitative study.

Participant Informed Consent Form

Study title:

Modeling Immersion Pathways in XR Cultural Heritage IP Narrative Experiences: An Integrated Approach Based on TAM, Experience Economy Theory, and Grounded Theory

- I have fully understood the contents of this study through the researcher's explanation and/or written materials.
- I have been informed of the possible risks (disadvantages) and benefits (advantages) associated with participation in this study.
- I voluntarily agree to participate in this study.
- I understand that I may refuse to participate or withdraw from the study at any time without giving a reason and without any disadvantage.
- I agree that, within the scope permitted by law and ethical review regulations, the researchers may collect and process the information I provide.
- I understand that I will receive a copy of this consent form.

Audio recording / video recording / photography: ☐ Agree / ☐ Do not agree

**Participant:**	(Signature)
**Date:**	Year ______ Month ______ Day ______
**Researcher:**	(Signature)
**Date:**	Year ______ Month ______ Day ______
